# Comparison of risankizumab and apremilast for the treatment of adults with moderate plaque psoriasis eligible for systemic therapy: results from a randomized, open-label, assessor-blinded phase IV study (IMMpulse)

**DOI:** 10.1093/bjd/ljad252

**Published:** 2023-07-25

**Authors:** Linda F Stein Gold, Jerry Bagel, Stephen K Tyring, H Chih-ho Hong, Lev Pavlovsky, Ronald Vender, Andreas Pinter, Adam Reich, Leonidas Drogaris, Tianshuang Wu, Manish Patel, Ahmed M Soliman, Huzefa Photowala, Vassilis Stakias, Sven Richter, Kim A Papp

**Affiliations:** Department of Dermatology, Henry Ford Hospital, Detroit, MI, USA; Psoriasis Treatment Center of New Jersey, East Windsor, NJ, USA; Department of Dermatology, Microbiology and Molecular Genetics and Internal Medicine, The University of Texas Medical School at Houston, Houston, TX, USA; Department of Dermatology and Skin Science, University of British Columbia, Vancouver, BC, Canada; Probity Medical Research, Surrey, BC, Canada; Tel Aviv University, Tel Aviv, Israel; Rabin Medical Centre, Petah Tikva, Israel; Dermatrials Research, Hamilton, ON, Canada; Venderm Consulting, Hamilton, ON, Canada; Department of Dermatology, University Hospital Frankfurt am Main, Frankfurt am Main, Germany; Department of Dermatology, Institute of Medical Sciences, Medical College of Rzeszów University, Rzeszów, Poland; AbbVie Inc., North Chicago, IL, USA; AbbVie Inc., North Chicago, IL, USA; AbbVie Inc., North Chicago, IL, USA; AbbVie Inc., North Chicago, IL, USA; AbbVie Inc., North Chicago, IL, USA; AbbVie Inc., North Chicago, IL, USA; AbbVie Inc., North Chicago, IL, USA; Probity Medical Research and Alliance Clinical Trials, Waterloo, ON, USA; Division of Dermatology, Department of Medicine, University of Toronto, Toronto, ON, Canada

## Abstract

**Background:**

Treatment of psoriasis with risankizumab has demonstrated superior efficacy to other treatments, such as adalimumab, ustekinumab and secukinumab.

**Objectives:**

This study compared the efficacy and safety of risankizumab and apremilast in adults with moderate plaque psoriasis eligible for systemic therapy. It also evaluated the efficacy and safety of switching to risankizumab vs. continuing apremilast in patients who did not achieve ≥ 75% improvement in Psoriasis Area and Severity Index (PASI 75 nonresponders) after 16 weeks of treatment with apremilast.

**Methods:**

This 52-week, phase IV, multicentre, randomized, open-label, efficacy assessor-blinded study (NCT04908475) enrolled patients (aged ≥ 18 years) with a diagnosis of moderate chronic plaque psoriasis (≥ 6 months) and who were candidates for systemic therapy. The enrolled patients (randomized 1 : 2) received subcutaneous risankizumab (150 mg at weeks 0 and 4) or oral apremilast (30 mg twice daily). At week 16, all patients treated with apremilast were re-randomized (1 : 1) to risankizumab or apremilast, stratified by week-16 PASI 75 response. The co-primary outcomes in period A at week 16 were the achievement of ≥ 90% improvement in Psoriasis Area and Severity Index (PASI 90) and static Physician’s Global Assessment (sPGA) 0/1 with a two-grade or better improvement from baseline. At week 52, the primary endpoint in period B was the achievement of PASI 90 in PASI 75 nonresponders with apremilast at week 16. Safety was monitored throughout the study. All patients who received one dose of treatment were included in the efficacy and safety analysis.

**Results:**

At baseline, 118 and 234 patients were assigned to receive risankizumab and apremilast, respectively. At week 16, PASI 90 was achieved by 55.9% [95% confidence interval (CI) 47.0–64.9] and 5.1% (95% CI 2.3–8.0), and sPGA 0/1 by 75.4% (95% CI 67.7–83.2) and 18.4% (95% CI 13.4–23.3), respectively. In period B, among PASI 75 nonresponders with apremilast at week 16, 83 switched to risankizumab and 78 continued apremilast. At week 52, 72.3% (95% CI 62.7–81.9) who switched to risankizumab achieved PASI 90 vs. 2.6% (95% CI 0.0–6.1) who continued apremilast. The most frequent adverse events (reported in ≥ 5%) in risankizumab-treated patients were COVID-19 infection and nasopharyngitis. Diarrhoea, nausea and headache were most frequent among apremilast-treated patients.

**Conclusions:**

For patients with moderate psoriasis, treatment with risankizumab demonstrated superior efficacy to those treated with apremilast, including those who did not benefit from prior treatment with apremilast. The safety profile of risankizumab was similar to prior studies, and no new safety signals were identified. These results show that, compared with apremilast, risankizumab treatment can significantly improve clinical outcomes in systemic-eligible patients with moderate psoriasis.

## Teaser

Risankizumab vs. apremilast treatment resulted in higher PASI 90 and sPGA 0/1 at week 16 in patients with moderate psoriasis eligible for systemic therapy. Risankizumab treatment was safe, with no new safety signals.

## Graphical Abstract

**Graphical Abstract:**
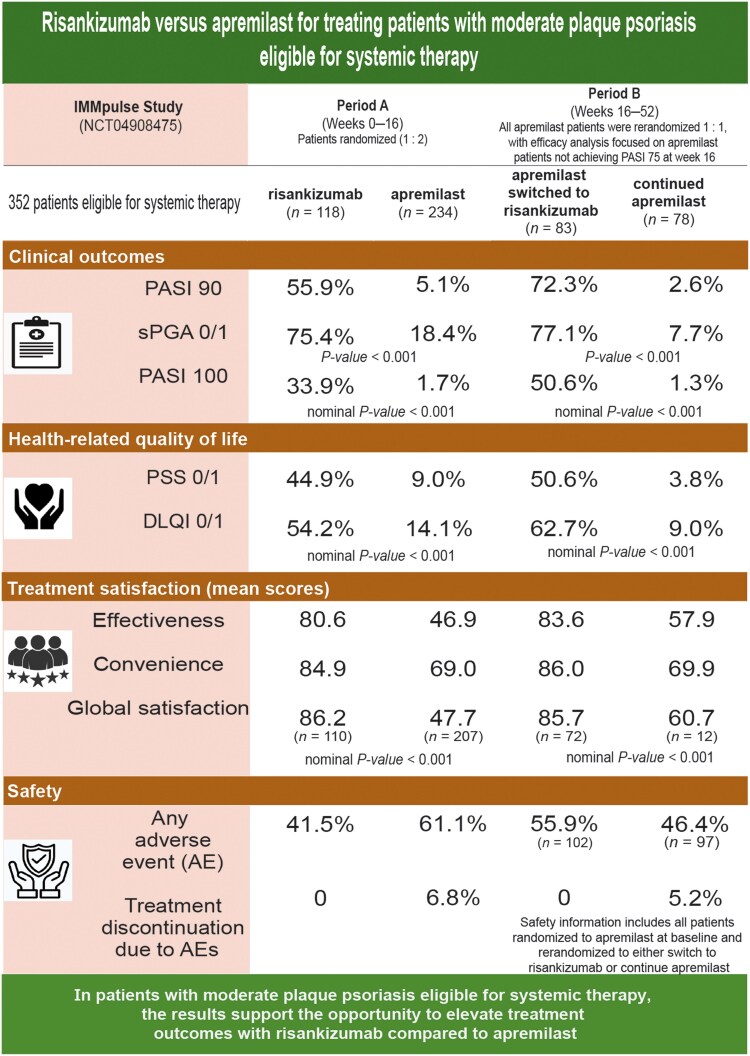

Plain language summary available online

Author Video: https://youtu.be/J1wo10vZF0o

**What is already known about this topic?**

- Risankizumab is a humanized IgG1 monoclonal antibody inhibitor of interleukin-23 approved for the treatment of moderate-to-severe plaque psoriasis and has demonstrated superior efficacy to other psoriasis treatments, such as adalimumab, ustekinumab and secukinumab.

**What does this study add?**

- Compared with apremilast treatment, treatment with risankizumab resulted in higher PASI 90 (≥ 90% improvement in Psoriasis Area and Severity Index) and static Physician’s Global Assessment (sPGA) 0/1 at week 16 in patients with moderate psoriasis eligible for systemic therapy.
- PASI 75 (≥ 75% improvement in PASI) nonresponders with apremilast at week 16, when switched to risankizumab, achieved higher rates of PASI 75, PASI 90 and sPGA 0/1 than those who continued apremilast.
- Risankizumab vs. apremilast treatment resulted in numerically higher overall treatment satisfaction and satisfaction with effectiveness and convenience.
- Risankizumab treatment was safe, with no new safety signals.

Psoriasis is a chronic, systemic, inflammatory condition associated with high individual and societal burdens, leading to significant disease concerns for patients.^1,2^ Despite effective treatments, many patients with psoriasis do not achieve high levels of skin clearance, as recommended in disease guidelines. Few achieve complete skin clearance and there remains an opportunity for improved patient satisfaction with treatment, less treatment discontinuation and reduced disease burden.^3–7^

Specific targeting of the regulatory cytokine interleukin (IL)-23 has been shown to play a critical role in treating psoriasis.^8,9^ Risankizumab is a humanized IgG1 monoclonal antibody that inhibits IL-23 by selectively binding to its p19 subunit. Studies have shown that risankizumab is well tolerated in patients with moderate-to-severe psoriasis, and many achieved 90% improvement in their disease. Risankizumab has also demonstrated superior efficacy to other psoriasis treatments, such as adalimumab, ustekinumab and secukinumab.^10–12^

Apremilast is approved for the treatment of psoriasis of all severities in the USA and moderate-to-severe plaque psoriasis in other countries, and is a commonly used oral systemic treatment for plaque psoriasis.^13,14^ In clinical studies, apremilast was safe and significantly reduced overall psoriasis severity and improved quality of life (QoL) outcomes in patients with mild-to-moderate plaque psoriasis.^15–17^ However, the efficacy profile of apremilast is lower than that of biologics, with ≥ 75% improvement in Psoriasis Area and Severity Index (PASI 75) and ≥ 90% improvement in PASI (PASI 90) rates at week 16 not exceeding 35% and 10%, respectively, in pivotal clinical studies.^16,17^

Evidence comparing risankizumab and apremilast in a clinical trial setting is currently unavailable. Herein, we report the results of the IMMpulse study (NCT04908475), which evaluated the efficacy and safety of risankizumab vs. apremilast in patients with moderate psoriasis eligible for systemic therapy. It also evaluated skin clearance outcomes after switching to risankizumab vs. continuing apremilast in patients who did not achieve a PASI 75 response (PASI 75 nonresponders) at week 16. Additionally, patient-reported outcomes (PROs) were evaluated, including health-related QoL (HRQoL), disease symptoms, treatment satisfaction, and work productivity and activity impairment.

## Patients and methods

### Patients

Patients aged ≥ 18 years were eligible if they had a diagnosis of moderate chronic plaque psoriasis (with or without psoriatic arthritis) for at least 6 months before enrolment and were candidates for systemic therapy. Moderate psoriasis was defined by a static Physician’s Global Assessment (sPGA) score of 3 based on a 5-point scale (0–4) at screening and the baseline visit; body surface area (BSA) involvement of ≥ 10% and ≤ 15%; and PASI ≥ 12. Complete eligibility criteria are described in Table S1 (see Supporting Information).

### Study design

IMMpulse was a phase IV, multicentre, randomized, open-label, efficacy assessor-blinded, active-comparator study. The study design comprised a screening period of up to 35 days, a 52-week treatment period and a follow-up telephone call for safety (Figure S1; see Supporting Information). The 52-week treatment duration included two periods: period A (weeks 0–16), which evaluated the superiority of risankizumab over apremilast; and period B (weeks 16–52), which evaluated the improvement of outcomes of switching to risankizumab vs. continuing apremilast in patients who were PASI 75 nonresponders with apremilast at week 16.

In period A, eligible patients were centrally randomized (1 : 2) at the baseline visit (day 1). They received either risankizumab 150 mg as a single subcutaneous (SC) injection or apremilast 30 mg orally twice daily (q12h). Randomization was stratified by baseline body weight (≤ 100 kg, > 100 kg) and prior exposure to any systemic and/or biologic treatment for psoriasis (0, ≥ 1). Apremilast administration began at baseline (day 1) based on the dose titration schedule from day 1 to 5 and continued with 30 mg q12h until the day before the week-16 visit, when all apremilast-treated patients were re-randomized. Risankizumab was administered at baseline (day 1) and week 4. The final efficacy evaluation for period A was at week 16.

In period B, patients initially randomized to risankizumab continued to receive risankizumab 150 mg as a single SC injection at weeks 16, 28 and 40. All patients initially randomized to apremilast were re-randomized at week 16 in a 1 : 1 ratio to receive either risankizumab 150 mg as a single SC injection at weeks 16, 20, 32 and 44 without washout, or apremilast 30 mg orally q12h up to week 52. Re-randomization was stratified by PASI 75 response to apremilast (PASI 75 responder or nonresponder) at week 16. Rescue with risankizumab was offered to patients who were re-randomized to apremilast and were PASI 50 (≥ 50% improvement in PASI) nonresponders at weeks 28 or 40. The final efficacy evaluation in period B was at week 52.

### Efficacy outcomes

In period A, the co-primary endpoints were the achievement of PASI 90 and sPGA 0/1 with at least a two-grade improvement from baseline at week 16. The ranked secondary endpoint was the achievement of PASI 75 at week 16. In period B, among PASI 75 nonresponders with apremilast at week 16, the primary endpoint was achievement of PASI 90 and the ranked secondary endpoints were achievement of PASI 75 and sPGA 0/1 with at least a two-grade improvement from baseline at week 52. Additional prespecified endpoints included the achievement of PASI 100 and sPGA 0.

### Patient-reported outcomes

Patient-reported measures included Psoriasis Symptoms Scale (PSS), Dermatology Life Quality Index (DLQI), Treatment Satisfaction Questionnaire for Medication version 9 (TSQM-9),^18,19^ and Work Productivity and Activity Impairment (WPAI). Achievement of PSS 0 and PSS 0/1, achievement of DLQI 0/1, achievement of minimally clinically important difference (MCID; reduction of ≥ 4 points) in DLQI scores among patients with baseline DLQI ≥ 4, changes from baseline in WPAI scores for impairments in work productivity and activities, and TSQM-9 scores at each postbaseline visit in period A were compared. Changes from the entry of period B among patients who were re-randomized at week 16 were also measured for all PROs.

### Safety outcomes

Safety was monitored throughout the study and is described in Table S2 (see Supporting Information). Treatment-emergent adverse events (TEAEs), serious adverse events (SAEs) and TEAEs leading to withdrawal were prespecified outcomes. TEAEs were graded as per the National Cancer Institute Common Terminology Criteria for Adverse Events Version 4.03.^20^

Prespecified areas of safety interest included major adverse cardiovascular events (MACE); serious infection; tuberculosis; fungal and opportunistic infections, including herpes zoster; malignancies; hypersensitivity reactions; and hepatic events. Independent cardiovascular adjudication committees adjudicated all observed cardiovascular, cerebrovascular and thrombotic events.

### Statistical analysis

The study was powered to detect the treatment differences between risankizumab and apremilast with respect to the co-primary endpoints in period A, as well as between apremilast switched to risankizumab and continued apremilast with respect to the primary endpoint in period B. It was assumed that 50% of apremilast PASI 75 nonresponders in the apremilast group switched to the risankizumab group and that 20% of apremilast PASI 75 nonresponders in the continued apremilast group would achieve PASI 90 at week 52 in period B, and that a sample size of 120 re-randomized apremilast PASI 75 nonresponders (60 patients per group) would have > 90% power to detect the treatment difference between apremilast switched to risankizumab and continued apremilast, using a χ^2^ test with a two-sided significance level of 0.05. Similarly, assuming that 55% of patients initially randomized to apremilast would be re-randomized at week 16 as apremilast PASI 75 nonresponders (as defined by PASI 75 response at week 16), the total sample size for the initial apremilast group was calculated as 220. Assuming that the treatment difference was expected to be at least 40% with respect to the co-primary endpoints of PASI 90 and sPGA of 0 or 1 at week 16 in period A, a total sample size of 330 patients (risankizumab, *n* = 110; apremilast, *n* = 220) should have provided > 90% power to detect the treatment difference between risankizumab and apremilast, using a χ^2^ test with a two-sided significance level of 0.05. Randomization was done using Interactive Response Technology.

Efficacy analyses were conducted in the intention-to-treat (ITT) population, which included all randomly assigned patients and all re-randomized patients at week 16. All statistical tests were performed at a two-sided alpha level of 0.05. The number and proportion of patients who achieved the endpoints were summarized within each treatment group. The proportion of responders for each co-primary endpoint in period A was compared between the risankizumab and apremilast groups [i.e. adjusted difference, 95% confidence interval (CI), *P*-value] using the Cochran–Mantel–Haenszel test adjusting for the stratification factors as per the initial randomization at baseline. Analysis of the primary endpoint in period B was conducted among PASI 75 nonresponder patients at week 16 based on the treatment group they were assigned to after re-randomization at week 16. The number and proportion of patients who achieved the endpoint were summarized within each treatment group. The proportion of responders was compared between the apremilast-switched-to-risankizumab and continued apremilast groups (i.e. adjusted difference, 95% CI and *P*-value) using the χ^2^ test. Missing data were handled by traditional nonresponder imputation and by incorporating multiple imputations due to the COVID-19 pandemic.

Overall type I error for period A was controlled by testing the co-primary endpoints, followed by the ranked secondary endpoint, in a hierarchical order. Control of overall type I error for period B was the same by testing the primary endpoint, followed by the ranked secondary endpoints, in a hierarchical order. No multiplicity adjustments were made for the additional prespecified efficacy endpoints.

Safety analyses were conducted on all patients receiving at least one treatment dose after randomization or re-­randomization. The data are reported as percentages and events per 100 patient-years for both risankizumab and apremilast between weeks 0 to 16 and 16 to 52, and for all patients who received risankizumab during the study.

## Results

### Patient disposition and baseline characteristics

**Figure 1:**
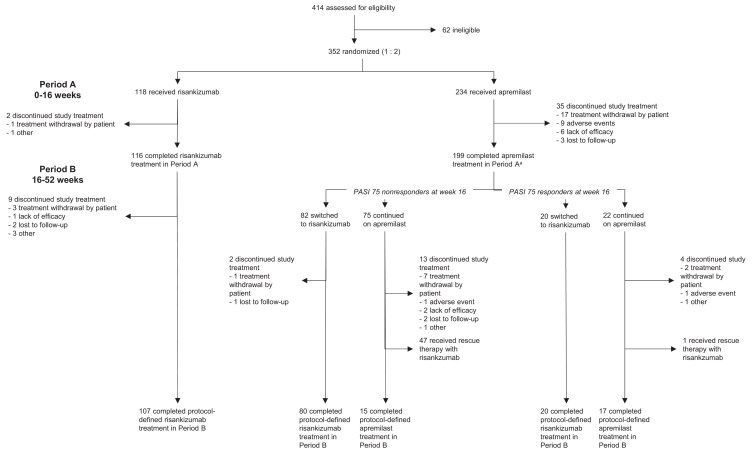
Flowchart showing the disposition of patients included in the phase IV IMMpulse study. ^a^In total, 203 patients randomized to apremilast at baseline were re-randomized at week 16; however, one patient re-randomized to switch to risankizumab and three patients re-randomized to continue apremilast were not dosed in period B – all four patients were considered apremilast discontinuations in period A.

Patients were enrolled from 51 sites across five countries: Canada, Germany, Israel, Poland and the USA. In period A, 352 patients were randomized and treated (118 received risankizumab; 234 received apremilast). The mean (SD) age of the study population was 46.0 (14.0) years; 65.6% were male, mean (SD) weight was 90.6 (22.1) kg and 32.1% received prior systemic and/or biologic therapy. Mean (SD) PASI was 14.5 (2.6) and mean (SD) BSA involvement was 13.1 (1.7)%. The demographics and baseline characteristics were evenly distributed between the two groups (Table 1). In period B, all apremilast-treated patients in period A were re-randomized (1 : 1) to either switch to risankizumab or continue with apremilast. Among apremilast-treated PASI 75 nonresponders in period A, 83 switched to risankizumab and 78 continued apremilast in period B. A total of 118 and 110 patients were included in the efficacy analyses of continuous treatment with risankizumab and apremilast from baseline through to week 52, respectively. Discontinuations during periods A and B are shown in Figure 1.

**Table 1:** Baseline demographics and clinical characteristics of the patients included in the phase IV IMMpulse study (randomization 1 : 2)

	Risankizumab (*n* = 118)	Apremilast (*n* = 234)
Age (years), mean (SD)	45.5 (13.6)	46.2 (14.3)
Age category (years)		
< 40	42 (35.6)	81 (34.6)
≥ 40	76 (64.4)	153 (65.4)
Sex		
Female	42 (35.6)	79 (33.8)
Male	76 (64.4)	155 (66.2)
Ethnicity		
Hispanic or Latino	7 (5.9)	23 (9.8)
Not Hispanic or Latino	111 (94.1)	211 (90.2)
Race		
White	98 (83.1)	209 (89.3)
Black or African American	5 (4.2)	9 (3.8)
Asian	12 (10.2)	14 (6.0)
American Indian or Alaska Native	0 (0)	1 (0.4)
Native Hawaiian or Other Pacific Islander	2 (1.7)	1 (0.4)
Multiple^a^	1 (0.8)	0
Weight (kg), mean (SD)	90.6 (19.1)	90.6 (23.5)
Weight (kg)^b^		
≤ 100	87 (73.7)	169 (72.2)
>100 kg	31 (26.3)	65 (27.8)
PASI, mean (SD)	14.5 (2.5)	14.5 (2.6)
BSA involvement (%), mean (SD)	13.1 (1.7)	13.1 (1.7)
sPGA categories		
≤ 2	0 (0)	1 (0.4)
3	118 (100)	232 (99.1)
4	0	1 (0.4)
PSS, mean (SD)	8.8 (3.4)	9.0 (3.5)
DLQI, mean (SD)	12.6 (6.9)	12.7 (7.1)
At least one medication prior to treatment	111 (94.1)	221 (94.4)
Prior systemic and/or biologic treatment ≥ 1^b^	37 (31.4)	76 (32.5)
Duration of plaque psoriasis (years), mean (SD)	18.5 (11.8)	17.8 (13.6)

Data are presented as *n* (%) unless otherwise stated. Race and ethnicity data were self-reported. BSA, body surface area; DLQI, Dermatology Life Quality Index; PASI, Psoriasis Area and Severity Index; PSS; Psoriasis Symptoms Scale; sPGA, static Physician’s Global Assessment. ^a^Patients who chose more than one category were placed in the ‘multiple’ category. ^b^Stratification factors for randomization.

In addition, at week 16, among all patients who were treated with apremilast at baseline, 42 achieved a PASI 75 response, of whom 20 switched to risankizumab and 22 continued apremilast in period B.

### Efficacy

**Figure 2:**
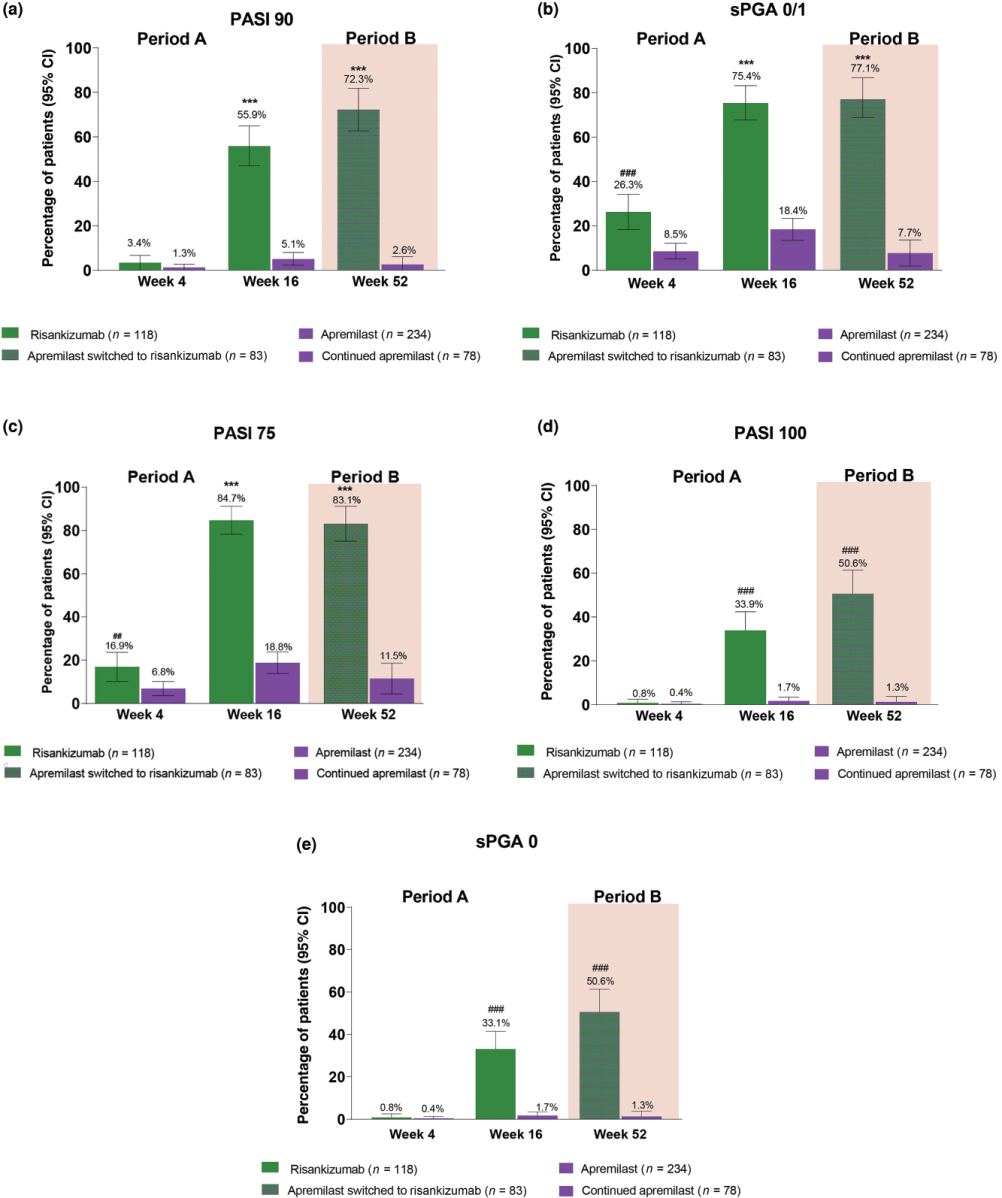
Co-primary, secondary and additional endpoints in the phase IV IMMpulse study. Proportion of patients achieving (a) ≥ 90% improvement in Psoriasis Area and Severity Index (PASI 90); (b) static Physician’s Global Assessment (sPGA) 0/1; (c) ≥ 75% improvement in PASI (PASI 75); (d) complete resolution of psoriatic lesions (PASI 100); and (e) sPGA 0 at weeks 4 and 16 (period A) and at week 52 (period B) after either switching to risankizumab or continuing apremilast in apremilast-treated patients not achieving PASI 75 at week 16. All error bars are present but may not be visible. In period A the proportion of responders for each co-primary endpoint was compared between the risankizumab and apremilast groups using the Cochran–Mantel–Haenszel test adjusting for stratification factors [baseline body weight (≤ 100 kg, > 100 kg) and prior exposure to any systemic and/or biological treatment for psoriasis (0, ≥ 1)]. In period B the proportion of responders was compared between the apremilast-switched-to-risankizumab and continued apremilast groups using the χ^2^ test. Nonresponder imputation incorporated multiple imputations to handle missing data only due to the COVID-19 pandemic. The period A intention-to-treat (ITT) population included all patients randomly assigned to receive risankizumab or apremilast from baseline until week 16; the period B ITT population included patients who were randomized to apremilast in period A, failed to achieve PASI 75 at week 16 and either switched to risankizumab or continued with apremilast in period B. *P < 0.05, **P < 0.01 and ***P < 0.001; ^#^*P* < 0.05 and ^###^*P* < 0.001 (nominally significant and not controlled for multiplicity). CI, confidence interval.

This study achieved all primary and ranked secondary endpoints [Figure 2; Figure S2 (see Supporting Information)]. In period A, the proportion of patients achieving the co-primary endpoints of PASI 90 and sPGA 0/1 was significantly higher in the risankizumab group: 55.9% (95% CI 47.0–64.9) of patients achieved PASI 90 and 75.4% (95% CI 67.7–83.2) achieved sPGA 0/1 with risankizumab compared with 5.1% (95% CI 2.3–8.0) and 18.4% (95% CI 13.4–23.3) with apremilast (both *P* < 0.001), respectively. The proportion of patients achieving the ranked secondary endpoint of PASI 75 at week 16 was also significantly higher in risankizumab- compared with apremilast-treated patients [84.7% (95% CI 78.–91.2) vs. 18.8% (95% CI 13.8–23.8); *P* < 0.001]. Additionally, complete clearance of psoriatic lesions [100% improvement in PASI (PASI 100) and sPGA 0] was achieved by higher proportions of patients treated with risankizumab vs. apremilast. PASI 100 and sPGA 0 were achieved by 33.9% (95% CI 25.4–42.4) and 33.1% (95% CI 24.6–41.5) on risankizumab, and 1.7% (95% CI 0.0–3.4) on apremilast for both, respectively.

Among apremilast PASI 75 nonresponders in period A who were re-randomized to risankizumab, significantly higher proportions of patients achieved PASI 90 compared with those reassigned to continue treatment with apremilast [72.3% (95% CI 62.7–81.9) vs. 2.6% (95% CI 0.0–6.1); *P* < 0.001] in period B. Similarly, a significantly higher proportion of patients re-randomized to risankizumab achieved PASI 75 [83.1% (95% CI 75.1–91.2) vs. 11.5% (95% CI 4.4–18.6)] and sPGA 0/1 [77.1% (95% CI 68.1–86.1) vs. 7.7% (95% CI 1.8–13.6)] at week 52 compared with patients who continued apremilast (*P* < 0.001). Around half (50.6%; 95% CI 39.8– 61.4) of patients who switched to risankizumab achieved PASI 100 and sPGA 0 each, while 1.3% (95% CI 0.0–3.8) achieved both PASI 100 and sPGA 0 in period B with continued apremilast.

**Figure 3:**
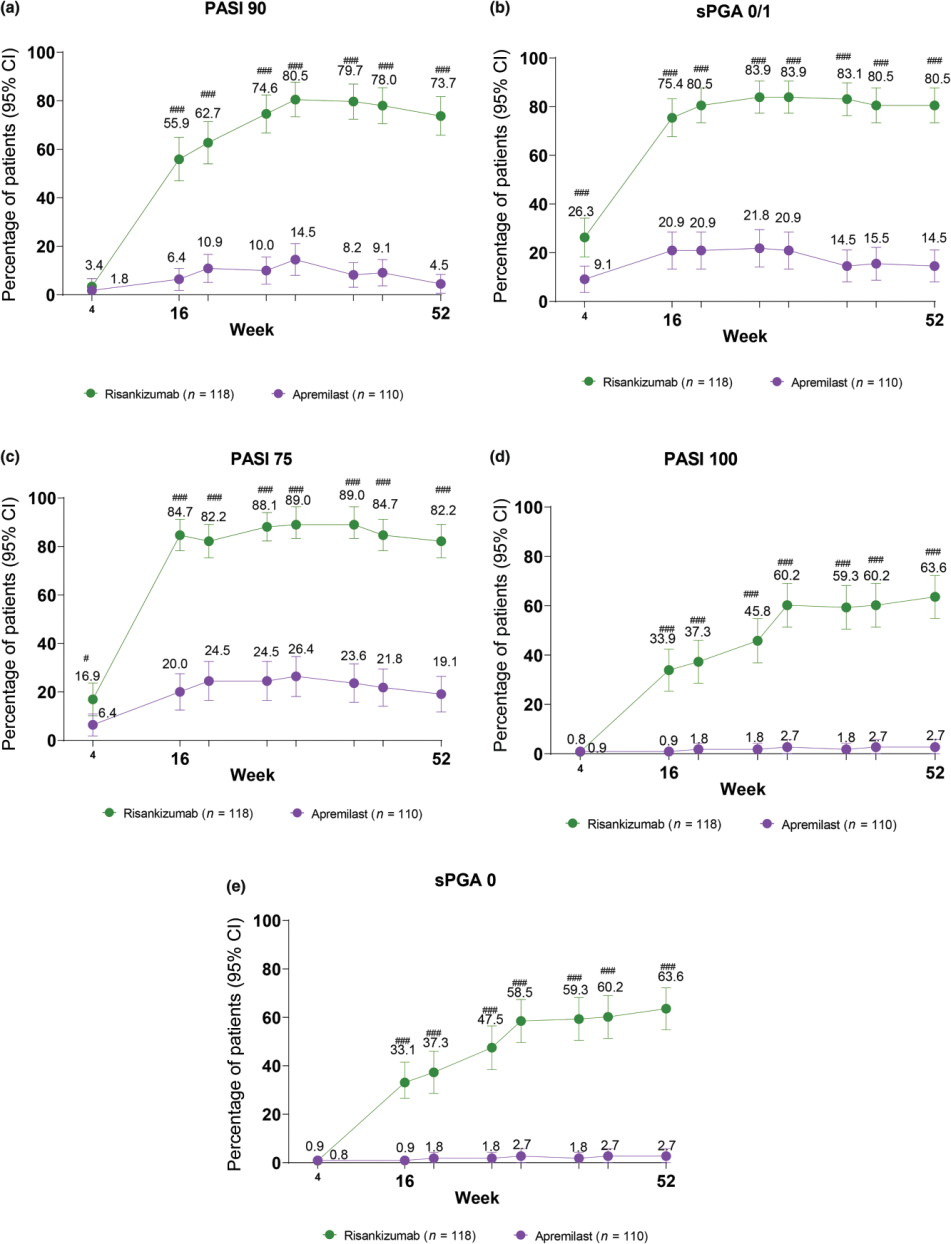
Skin clearance endpoints of patients who were treated with risankizumab and apremilast for the entire 52-week study period of IMMpulse. The proportion of patients achieving (a) ≥ 90% improvement in Psoriasis Area and Severity Index (PASI 90); (b) static Physician’s Global Assessment (sPGA) 0/1 (sPGA 0/1); (c) ≥ 75% improvement in PASI (PASI 75); (d) complete resolution of psoriatic lesions (PASI 100); and (e) sPGA 0 from week 4 to week 52 in patients receiving risankizumab or apremilast for the full study period from baseline to week 52. All error bars are present but may not be visible. Nonresponder imputation incorporated multiple imputations to handle missing data only due to the COVID-19 pandemic. The long-term intention-to-treat population included all patients randomly assigned to receive risankizumab from baseline, and all patients randomly assigned to receive apremilast from baseline and then re-randomized to continue apremilast at week 16, as well as half of the apremilast patients who discontinued from the study in period A at the time of the week-16 interim database lock in June 2022. ^#^*P* < 0.05 and ^###^*P* < 0.001 (nominally significant and not controlled for multiplicity).

In patients who continued baseline treatment beyond week 16 (Figure 3), continuing risankizumab achieved numerically higher (nominal *P* < 0.001) PASI 90 (73.7%; 95% CI 65.8–81.7), PASI 75 (82.2%; 95% CI 75.3–89.1) and sPGA 0/1 (80.5%; 95% CI 73.4–87.7) response rates than those who continued apremilast [4.5% (95% CI 0.7–8.4); 19.1% (95% CI 11.7–26.4); and 14.5% (95% CI 8.0–21.1), respectively] at week 52. Absolute skin clearance (PASI 100 and sPGA 0) were achieved by more patients (nominal *P* < 0.001) on risankizumab [63.6% (95% CI 54.9–72.2) for both outcomes] than on apremilast [2.7% (95% CI 0.0–5.8) for both outcomes].

**Figure 4:**
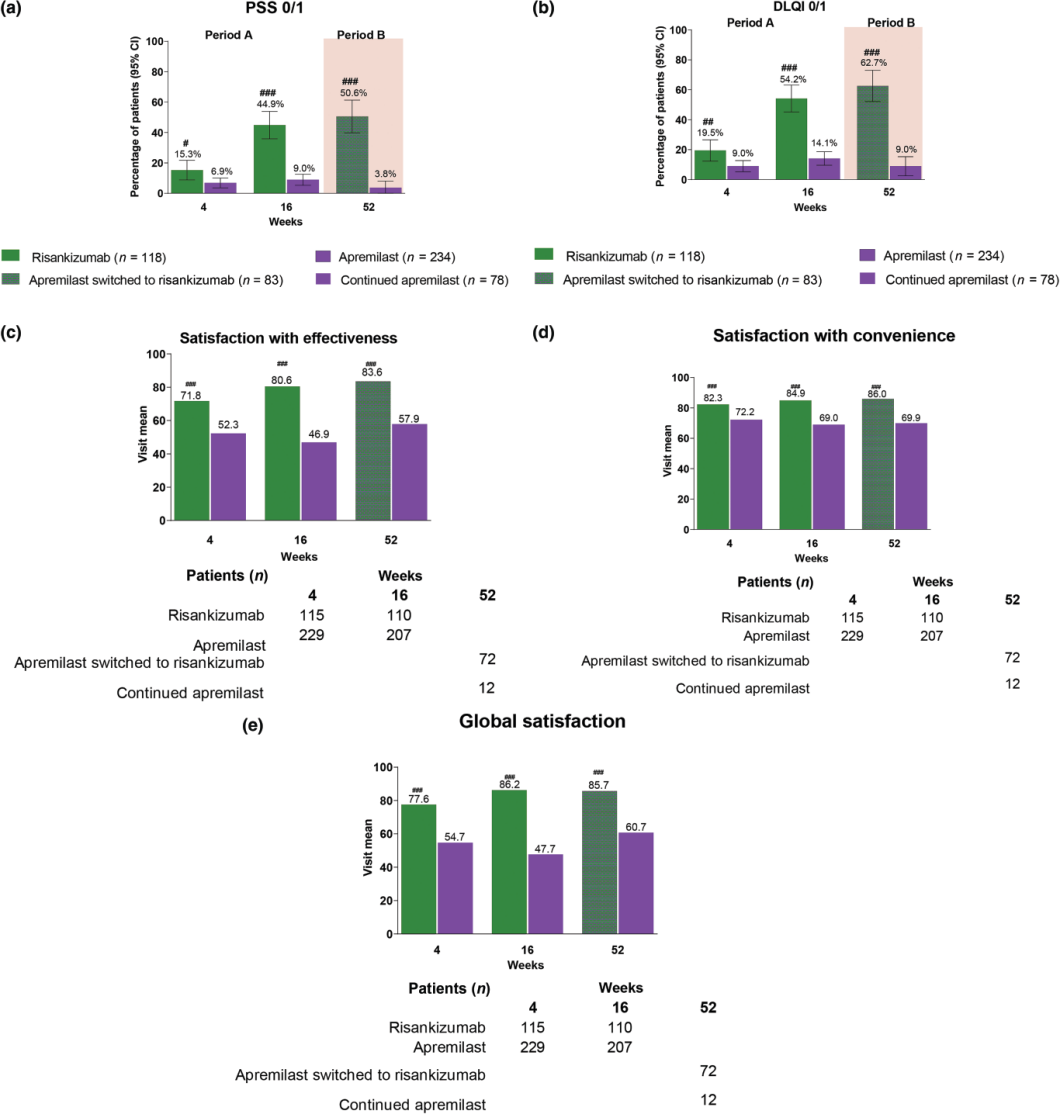
Psoriasis symptoms, health-related quality of life and treatment satisfaction. The proportion of patients achieving (a) Psoriasis Symptoms Scale (PSS) 0/1; (b) Dermatology Life Quality Index (DLQI) 0/1; and visit mean (c) Treatment Satisfaction Questionnaire for Medication version 9 (TSQM) global satisfaction, (d) TSQM effectiveness and (e) TSQM convenience at weeks 4 and 16 (period A) and at week 52 (period B) after either switching to risankizumab or continuing apremilast in apremilast-treated patients not achieving ≥ 75% improvement in Psoriasis Area and Severity Index (PASI 75) at week 16. The period A intention-to-treat (ITT) population included all patients randomly assigned to receive risankizumab or apremilast from baseline until week 16; the period B ITT population included patients who were randomized to apremilast in period A, failed to achieve PASI 75 at week 16, and either switched to risankizumab or continued with apremilast in period B. ^#^*P* < 0.05 and ^###^*P* < 0.001 (nominally significant and not controlled for multiplicity). Nonresponder imputation incorporating multiple imputations to handle missing data due to the COVID-19 pandemic was used for binary endpoints. For TSQM, a mixed model for repeated measures analysis was used; treatment, visit and treatment × visit interaction was used in the model for variance estimation.

In addition, patients reported greater benefits to their psoriasis symptoms and HRQoL with risankizumab [Figure 4a, b; Figure S3a, b (see Supporting Information)]. In period A, a greater proportion of patients achieved PSS 0/1 [44.9% (95 CI 35.9–53.9) vs. 9.0% (95% CI 5.3–12.6)], DLQI 0/1 [54.2% (95% CI 45.2–63.2) vs. 14.1% (95% CI 9.6–18.6)], PSS 0 [29.7% (95% CI 21.4–37.9) vs. 3.0% (95% CI 0.8–5.2)] and DLQI MCID [82.4% (95% CI 75.2–89.6) vs. 52.6% (95% CI 45.9–59.3)] than apremilast-treated patients, respectively (nominal *P* < 0.001).

In patients who continued the same period A treatment beyond week 16, a greater proportion of patients on risankizumab achieved PSS 0/1 (60.2%; 95% CI 51.3–69.0), DLQI 0/1 (66.1%; 95% CI 57.6–74.6), PSS 0 (46.6%; 95% CI 37.6–55.6) and DLQI MCID (76.9%; 95% CI 68.9–84.8) at week 52 – all numerically higher than the proportions observed at week 16 for risankizumab-treated patients (all nominal *P* < 0.001). These were numerically higher than the PSS 0/1 (4.5%; 95% CI 0.7–8.4), DLQI 0/1 (9.1%; 95% CI 3.7–14.5), PSS 0 (0%) and DLQI MCID (15.8%; 95% CI 8.7–23.0) observed at week 52 in patients treated with continuous apremilast from baseline (Figure S4; see Supporting Information).

Improvements in TSQM-9 are shown in Figure 4(c–e). In period A, compared to apremilast-treated patients, risankizumab-treated patients reported higher satisfaction with effectiveness (Δ 34.3, 95% CI 28.3–40.3), satisfaction with convenience (Δ 16.3, 95% CI 11.8–20.7) and global satisfaction [adjusted difference (Δ) 39.8, 95% CI 34.2–45.5]. Compared with apremilast-treated patients, those treated with risankizumab also reported greater reductions in overall work impairment (Δ –12.0%, 95% CI –18.0 to –6.1) and activity impairment (Δ –15.1%, 95% CI –20.1 to –10.1) from baseline. Patients who switched from apremilast to risankizumab in period B recorded improved treatment satisfaction with effectiveness, convenience and global satisfaction (Figure S3c–e), and overall work and activity impairment in period B (Figure S5; see Supporting Information).

Efficacy observations in apremilast-treated patients at baseline who achieved a PASI 75 response at week 16 and either switched to risankizumab or continued apremilast in period B were similar to those in the overall ITT population (Table S3; see Supporting Information).

### Safety

Safety data from periods A and B are presented in Table 2. In period A, TEAEs were reported in 41.5% of patients treated with risankizumab vs. 61.1% treated with apremilast, with 5.1% vs. 41.9% being possibly related to the study treatment. Severe AEs (0.8% vs. 3.8%) and SAEs (0.8% vs. 1.7%) were lower with risankizumab vs. apremilast treatment. In period A, there was no treatment discontinuation due to AEs with risankizumab, while 6.8% experienced an AE that led to discontinuation of apremilast. The most frequently reported TEAE (occurring in ≥ 5% of patients in any treatment group) with risankizumab was COVID-19 infection (11.0%); for apremilast, diarrhoea (20.1%), nausea (17.5%), headache (11.5%) and COVID-19 infection (6.8%) were the most frequently reported TEAEs.

**Table 2:** Overview of treatment-emergent adverse events (TEAEs) in periods A and B of the phase IV IMMpulse study

TEAEs	Period A (baseline to week 16)			
	Risankizumab 150 mg (*n* = 118), *n* (%)	Risankizumab 150 mg (*n* = 118), events (events/100 PY)^a^	Apremilast 30 mg (*n* = 234), *n* (%)	Apremilast 30 mg (*n* = 234, events (events/100 PY)^b^
AE	49 (41.5)	81 (226.3)	143 (61.1)	308 (457.7)
AE with reasonable possibility of being related to study treatment^c^	6 (5.1)	9 (25.1)	98 (41.9)	169 (251.1)
Severe AE	1 (0.8)	1 (2.8)	9 (3.8)	17 (25.3)
SAE	1 (0.8)	1 (2.8)	4 (1.7)	4 (5.9)
AE leading to discontinuation of study drug	0 (0)	0 (0)	16 (6.8)	34 (50.5)
AE leading to death	0 (0)	0 (0)	0 (0)	0 (0)
TEAEs reported in ≥ 5% of patients				
Diarrhoea	1 (0.8)	1 (2.8)	47 (20.1)	50 (74.3)
Nausea	0 (0)	0 (0)	41 (17.5)	45 (66.9)
Headache	3 (2.5)	3 (8.4)	27 (11.5)	28 (41.6)
COVID-19 infection	13 (11.0)	13 (36.3)	16 (6.8)	16 (23.8)
	**Period B (weeks 16–52, all patients randomized to apremilast at baseline)**			
	**Apremilast switched to risankizumab (*n* = 102), *n* (%)**	**Apremilast switched to risankizumab (*n* = 102), events (events/100 PY)^d^**	**Continued apremilast (*n* = 97), *n* (%)**	**Continued apremilast (*n* = 97), events (events/100 PY)^e^**
AE	57 (55.9)	123 (141.5)	45 (46.4)	91 (226.4)
AE with reasonable possibility of being related to study treatment^c^	11 (10.8)	13 (15.0)	14 (14.4)	19 (47.3)
Severe AE	3 (2.9)	5 (5.8)	2 (2.1)	2 (5.0)
SAE	3 (2.9)	6 (6.9)	2 (2.1)	2 (5.0)
AE leading to discontinuation of study drug	0 (0)	0 (0)	5 (5.2)	5 (12.4)
AE leading to death	0 (0)	0 (0)	0 (0)	0 (0)
TEAEs reported in ≥ 5% of patients				
COVID-19 infection	12 (11.8)	12 (13.8)	14 (14.4)	14 (34.8)
Nasopharyngitis	10 (9.8)	11 (12.7)	8 (8.2	10 (24.9)
URTI	6 (5.9)	8 (9.2)	3 (3.1)	3 (7.5)
Headache	5 (4.9)	6 (6.9)	4 (4.1)	4 (10.0)

AE, adverse event; PY, person-years; SAE, serious AE; URTI, upper respiratory tract infection. ^a^PY = 35.8; ^b^PY = 67.3; ^c^investigators assessed AE to be possibly related to study treatment; ^d^PY = 86.9; ^e^PY = 40.2.

In period B, among all patients who were randomized to apremilast at baseline, 102 switched to risankizumab and 97 continued apremilast, irrespective of their PASI 75 response at week 16. In this population, TEAEs were reported in 55.9% of patients who switched from apremilast to risankizumab and in 46.4% of patients who continued apremilast; 10.8% of TEAEs were possibly related to risankizumab and 14.4% related to apremilast treatment. Severe AEs (2.9% vs. 2.1%) and SAEs (2.9% vs. 2.1%) were slightly higher with risankizumab treatment during this period. The most frequently reported TEAEs (occurring in ≥ 5% of patients in any treatment group) during period B were COVID-19 infection (11.8% vs. 14.4%), nasopharyngitis (9.8% vs. 8.2%) and upper respiratory tract infection (5.9% vs. 3.1%) among patients who switched to risankizumab vs. those who continued apremilast. No deaths were reported in the study.

In relation to TEAEs of special interest, there were no reports of MACE, malignancy, serious infections or serious hypersensitivity reported with risankizumab treatment during period A (Table S4; see Supporting Information). In the apremilast group, there was a single event each for adjudicated and extended MACE and serious infection.

In period B, among patients who switched to risankizumab, there were three events of hypersensitivity (two due to allergic rhinitis), of which there was one event of serious hypersensitivity reaction with no reasonable possibility of a causal association to risankizumab, as determined by the investigator. In addition, there was one nonserious hepatic event with no reasonable possibility of a causal association to risankizumab, as determined by the investigator. One event of extended MACE was reported among patients who continued with apremilast. Two events of hypersensitivity and one event of malignant tumour were also reported during this period with apremilast treatment.

TEAEs among all patients treated with risankizumab are listed in Table S5 (see Supporting Information). Sixty-two per cent of patients reported an AE, of which 10.4% were possibly related to risankizumab treatment. One AE led to the discontinuation of risankizumab treatment (due to lymphoma) with no reasonable possibility it was caused by the treatment. No deaths were reported.

## Discussion

In this study, patients with moderate plaque psoriasis eligible for systemic therapy and treated with risankizumab showed superior efficacy outcomes to those treated with apremilast. All primary endpoints and ranked secondary endpoints in both study periods were achieved. The responses noted with risankizumab were rapid and maintained throughout the 52-week study. Patients who switched to risankizumab after inadequate response to apremilast at week 16 also experienced significantly improved efficacy outcomes. Complete resolution of psoriatic lesions (PASI 100, sPGA 0) was observed with risankizumab treatment. At week 52, 63.6% of patients who received continuous risankizumab from baseline achieved PASI 100 and sPGA 0 vs. only 2.7% of patients receiving continuous apremilast.

Psoriasis has a negative impact on patients’ HRQoL, affecting their daily activities.^21,22^ In addition to improved clinical efficacy outcomes, patients receiving risankizumab reported improvements in HRQoL. At baseline, the mean DLQI score was 12.7, indicating a very large effect of psoriasis on patients’ lives, similar to that observed in other studies in people with moderate-to-severe psoriasis.^11,23^ At week 16, 44.9% of patients receiving risankizumab reported no impact of psoriasis on HRQoL (PSS 0/1) vs. only 9% with apremilast. Loss of work productivity contributes to a significant economic burden in patients with psoriasis.^24^ Risankizumab-treated patients reported improved work productivity and reduced activity impairment vs. those treated with apremilast.

There is a correlation between decreased patient treatment satisfaction and poor adherence to psoriasis treatment.^25^ Risankizumab-treated patients reported greater treatment satisfaction, with increased satisfaction in all three domains of satisfaction with effectiveness, satisfaction with convenience and global satisfaction vs. apremilast-treated patients. Many patients may prefer oral administration of treatment; however, patients with moderate psoriasis have reported highly valuing HRQoL improvement and maintenance of response, and dosing frequency was the most important attribute driving treatment choice.^26,27^ Hence, the high efficacy and improvement in HRQoL with risankizumab administered quarterly may be an important patient consideration when making treatment decisions.

Risankizumab was well tolerated, and the observed TEAEs were consistent with previous clinical studies.^10,11,27^ No new safety concerns were identified in this study population of patients with systemic-eligible moderate psoriasis. Additionally, no new safety concerns were identified in risankizumab-treated patients who switched from apremilast to risankizumab without a washout period. In period A, the rates of TEAEs, AEs related to study treatment, severe AEs, SAEs and AEs leading to treatment discontinuation were numerically higher with apremilast than with risankizumab treatment. A high proportion of apremilast-treated patients discontinued treatment in the first 16 weeks of therapy. The percentage and the event rates per 100 person-years of exposure are consistent with those observed in previous apremilast clinical trials, including the high frequency of nausea, diarrhoea and vomiting in the apremilast group.^28^ This contrasts with the risankizumab experience in this study, with nearly all patients receiving treatment up to week 16 and none discontinuing risankizumab due to an AE.

At week 16, the PASI 90 response rates were lower than in previous studies of risankizumab and apremilast in patients with moderate-to-severe psoriasis.^11,16,17^ One consideration is the known nonlinearity between PASI and disease severity, which may result in lower relative improvements in patient populations with lower PASI scores at baseline.^28,29^

The risankizumab PASI 100 results in the IMMpulse study at weeks 16 and 52 are similar to those of previous trials, with approximately 33.9% and 63.6% of patients achieving absolute skin clearance at weeks 16 and 52, respectively. These rates of skin clearance are important clinically when considering the low likelihood of PASI 100 associated with therapies such as apremilast, commonly used in systemic-eligible patients with moderate psoriasis.^16,17^

A potential study limitation is the open-label design; however, efficacy assessments were conducted in a blinded manner throughout the study. A blinded assessor design has been consistently shown to provide reliable comparative results without the burden of a double-blind design for phase IV studies. This approach has been used in several recent studies comparing active treatments for plaque psoriasis and psoriatic arthritis.^12,30,31^ There may be a bias toward the retention of patients with improved efficacy and without safety concerns. Besides the co-primary endpoints and ranked secondary endpoint in period A, and the primary endpoint and ranked secondary endpoints in period B, all other comparisons were performed without adjusting for multiplicity and should be interpreted with caution. In addition, the study lasted for a year, and psoriasis is a chronic condition that warrants long-term follow-up. Future studies should also enrol patients from ethnic minorities and diverse geographical locations, to enhance the generalizability of the results and to ensure a broader representation of populations affected by the condition.

These results show that risankizumab, administered every 12 weeks, can be used to achieve high rates of clinical response, with favourable safety and tolerability vs. apremilast. Improved PROs measuring HRQoL, treatment satisfaction and work productivity further support the favourability of risankizumab relative to apremilast in the systemic-­eligible moderate psoriasis patient population.

## Supplementary Material

ljad252_Supplementary_Data

## Data Availability

AbbVie is committed to responsible data sharing regarding the clinical trials we sponsor. This includes access to anonymized, individual and trial-level data (analysis datasets), as well as other information (e.g. protocols, clinical study reports and analysis plans), as long as the trials are not part of an ongoing or planned regulatory submission. This includes requests for clinical trial data for unlicensed products and indications. This clinical trial data can be requested by any qualified researchers who engage in rigorous, independent scientific research and will be provided following review and approval of a research proposal, statistical analysis plan and execution of a data sharing agreement. Data requests can be submitted at any time after approval in the USA and Europe, and after acceptance of this manuscript for publication. The data will be accessible for 12 months, with possible extensions considered. For more information on the process or to submit a request, visit the following link: https://vivli.org/ourmember/abbvie/ then select ‘Home’.
