# Supplementary material for: Comparison of risankizumab and apremilast for the treatment of adults with moderate plaque psoriasis eligible for systemic therapy: results from a randomized, open-label, assessor-blinded phase IV study (IMMpulse)
Source: Br J Dermatol. 2023 Jul 25;189(5):540–52. doi: 10.1093/bjd/ljad252 (PMC13077218; doi:10.1093/bjd/ljad252)
Supplement: ljad252_Supplementary_Data [file ljad252_supplementary_data.docx]

**Comparison of risankizumab and apremilast for the treatment of adult patients with moderate plaque psoriasis eligible for systemic therapy: results from a randomised, open-label, assessor-blinded phase IV (IMMpulse) study**

**Supplemental Appendix**

Figure S1 IMMpulse study design (NCT04908475)


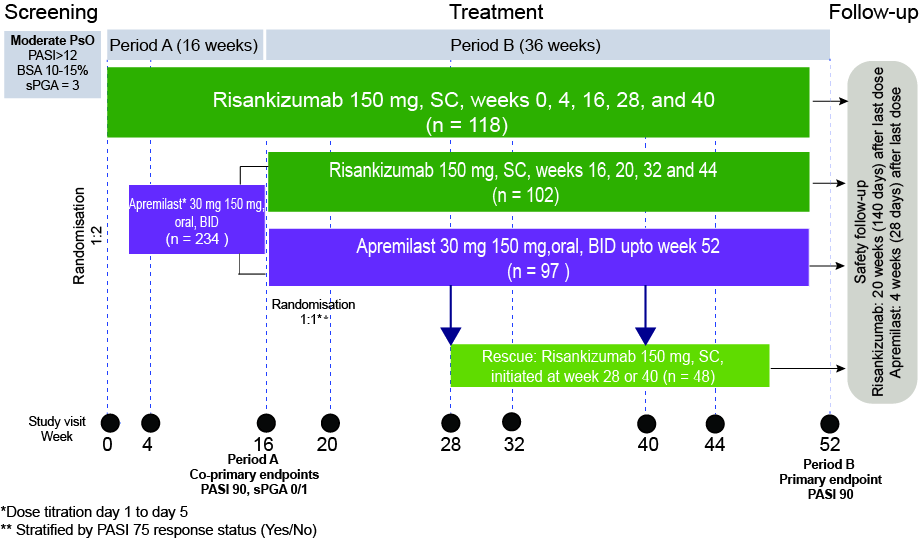


BID, twice daily; BSA, Body Surface Area; PASI, Psoriasis Area Severity Index; SC, subcutaneous, sPGA, static Physician's Global Assessment

Figure S2 Primary, secondary, and additional endpoints in Period B


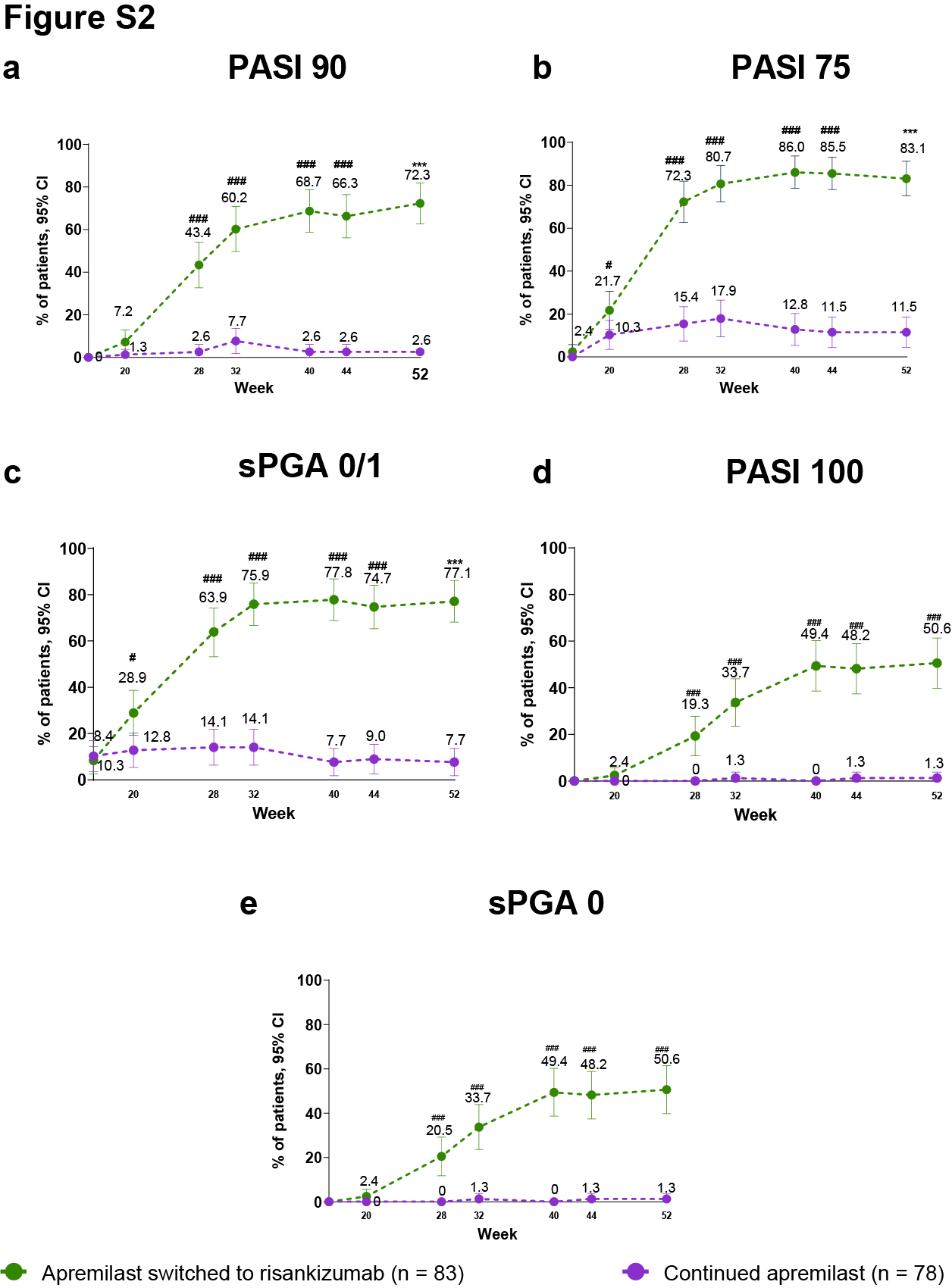


PASI, Psoriasis Area and Severity Index; sPGA, static Physician's Global Assessment.

The proportion of patients in Period B achieving the following outcomes: a. PASI 90, b. sPGA 0/1, c. PASI 75, d. PASI 100, e. sPGA 0 at each timepoint from week 16 to 52.

Period B included patients who were randomised to apremilast in Period A, failed to achieve PASI 75 at week 16 and either switched to risankizumab or continued with apremilast in Period B.

All error bars are present but may not be visible.

*P-value* for interaction between subgroup and treatment was calculated using a logistic regression with visit measurement at week 52 as response variable, with treatment, subgroup and treatment by subgroup interaction as factors. Logistic regression was based on non-responder imputation incorporating multiple imputation to handle missing data due to COVID-19 or non-responder imputation only if there were no missing data due to COVID-19. For multiple imputation data, one-sided *P-value* was calculated based on Student's t-distribution using the Chi-square test.

***, **, * Statistically significant at the 0.001, 0.01, and 0.05 level, respectively.

Figure S3 Psoriasis symptoms, health-related quality-of-life, and treatment satisfaction


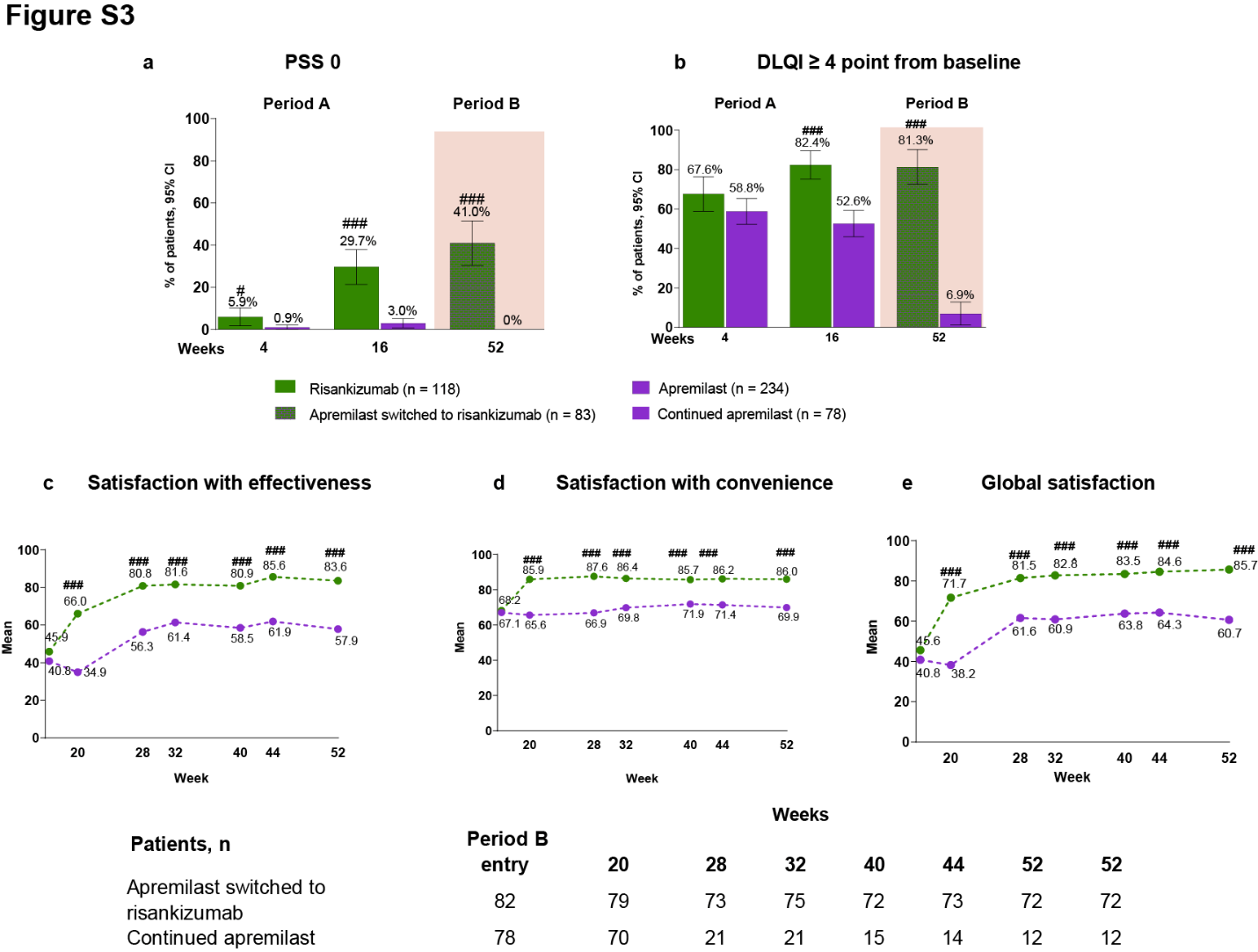


DLQI, Dermatology Life Quality Index; PSS, Psoriasis Symptoms Scale; TSQM, Treatment Satisfaction Questionnaire for Medication version 9.

The proportion of patients achieving a. PSS 0, b. DLQI 0 ≥4 point improvement from baseline at week 4 and week 16 (Period A) and at week 52 (Period B) after either switching to risankizumab or continuing apremilast in apremilast-treated patients not achieving PASI 75 at week 16.

Visit mean c. Satisfaction with effectiveness, d. Satisfaction with convenience, E. Global satisfaction in Period B after either switching to risankizumab or continuing apremilast in apremilast-treated patients not achieving PASI 75 at week 16.

All error bars are present but may not be visible.

Period A Intention-to Treat (ITT) population included all patients randomly assigned to receive risankizumab or apremilast from baseline until week 16; Period B ITT population included patients who were randomised to apremilast in Period A, failed to achieve PASI 75 at week 16 and either switched to risankizumab or continued with apremilast in Period B.

###, #, nominally significant at the 0.001 and 0.05 levels, respectively, and not controlled for multiplicity.

Non-responder imputation incorporating multiple imputations to handle missing data due to COVID-19 was used for binary endpoints. For TSQM, a MMRM analysis was used; treatment, visit, and treatment by visit interaction was used in the model for variance estimation.

Figure S4 Psoriasis symptoms and health-related quality-of-life among patients who were treated with risankizumab and apremilast for the 52-week study period


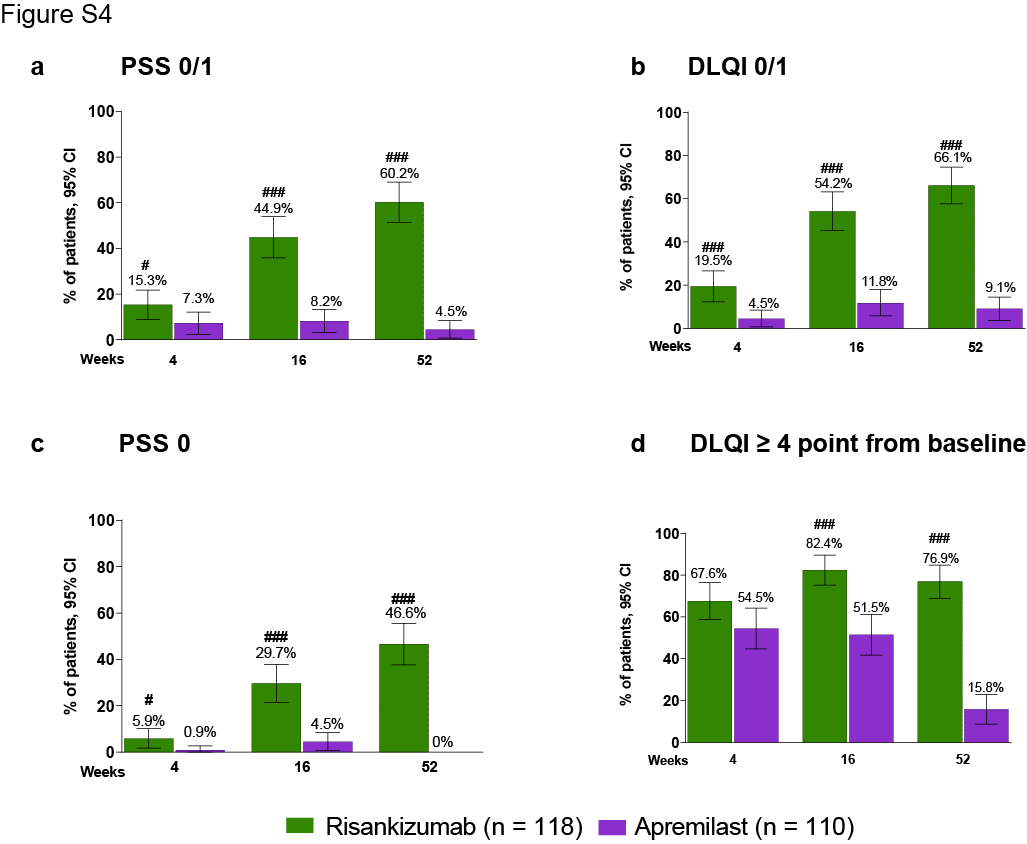


DLQI, Dermatology Life Quality Index; PSS, Psoriasis Symptoms Scale

The proportion of patients achieving a. PSS 0/1, b. DLQI 0/1, c. PSS 0, and d. DLQI 0 ≥4 point improvement from baseline at week 4, week 16 and week 52 in patients receiving risankizumab or apremilast for the full study period from baseline to week 52.

Non-responder imputation incorporated multiple imputations to handle missing data only due to COVID-19.

Long-term ITT population included all patients randomly assigned to receive risankizumab from baseline, and all patients randomly assigned to receive apremilast from baseline and then rerandomized to continue apremilast at week 16 as well as half of the apremilast patients who discontinued from the study in Period A at the time of the week 16 interim database lock in June 2022.

###, #, nominally significant at the 0.001, 0.05 level, respectively and not controlled for multiplicity.

Figure S5 Work Productivity and Activity Impairment (WPAI) - overall work impairment and activity impairment


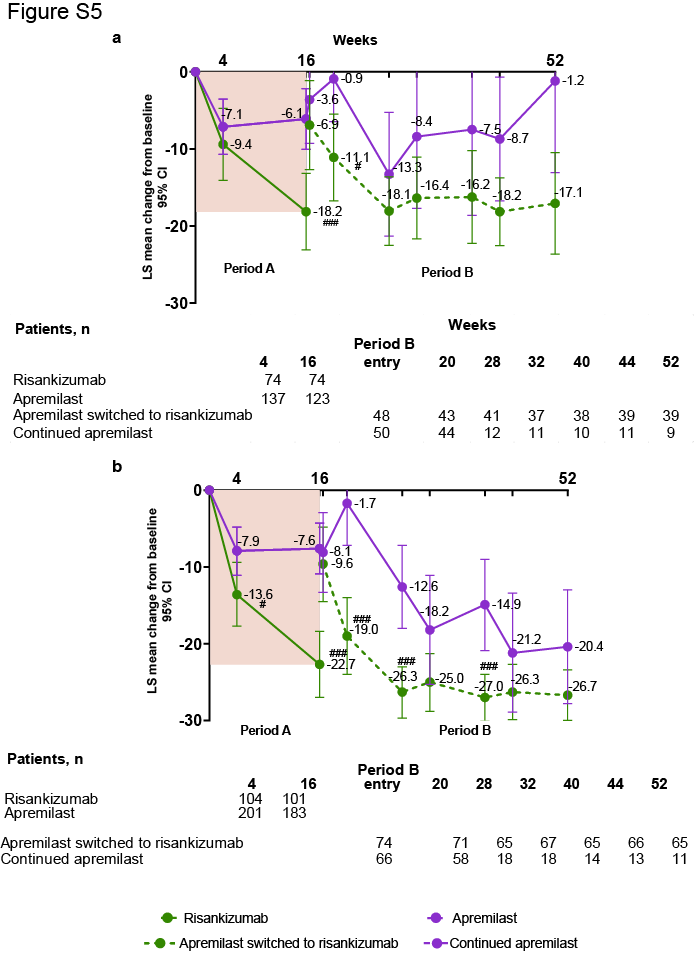


CI, confidence interval; LS, least squared

LS mean change improvement from baseline in a. WPAI overall work impairment, b. WPAI activity impairment at week 4 and 16 (Period A) and at Period B entry, week 20 to week 52 (Period B) after either switching to risankizumab or continuing apremilast in apremilats-treated patients not achieving PASI 75 at week 16.

Period A ITT population included all patients randomly assigned to receive risankizumab or apremilast from baseline until week 16; Period B ITT population included patients who were randomised to apremilast in Period A, failed to achieve PASI 75 at week 16, and either switched to risankizumab or continued with apremilast in Period B.

All error bars are present but may not be visible.

A MMRM analysis was used; treatment, visit, and treatment by visit interaction was used in the model for variance estimation. Baseline was defined as the last non-missing value prior to the first dose of study drug or randomisation. If no study drug was given, patients with non-missing baseline and at least one post-baseline value were included in the analysis.

###, #, nominally significant at the 0.001, 0.05 level, respectively and not controlled for multiplicity.

Table S1 Eligibility criteria of patients enrolled the IMMpulse study

| **Inclusion criteria** |
| --- |
| - Adult patients (≥ 18 years) were eligible if they had a diagnosis of moderate chronic plaque psoriasis (with or without psoriatic arthritis) for at least six months before enrollment and were candidates for systemic therapy. Moderate psoriasis was defined by static Physician's Global Assessment (sPGA) = 3 (moderate) based on a 5-point scale (0 to 4) at screening and the baseline visit and BSA involvement ≥ 10% and ≤ 15%, and PASI ≥ 12. - Patients must have met the following laboratory values within the screening period prior to the first dose of study drug: - Serum aspartate transaminase (AST) ≤ 2 × upper limit of normal (ULN) - Serum alanine transaminase (ALT) ≤ 2 × ULN - Serum total bilirubin ≤ 2.0 mg/dL; except for patients with isolated elevation of indirect bilirubin relating to Gilbert syndrome - Total white blood cell count ≥ 3,000/μL - Absolute neutrophil count ≥ 1,500/μL - Platelet count ≥ 100,000/μL - Haemoglobin ≥ 10 g/dL (100 g/L) - Estimated glomerular filtration rate by the Chronic Kidney Disease Epidemiology - Collaboration equation (eGFR [CKD-EPI]) ≥ 30 mL/min/1.73 m^2^ - Psoriasis (PsO) had inadequately controlled disease by topicals, phototherapy and/or systemic treatments - Patient was judged to be in good general health, as determined by the investigator based upon the results of a medical history, physical examination, laboratory profile, and a 12-lead electrocardiogram (ECG) performed during the screening period - For all females of child-bearing potential; a negative serum pregnancy test at the screening visit and a negative urine pregnancy test at baseline prior to the first dose of study drug was required |
| **Exclusion criteria** |
| - Patients must not have had any form of PsO other than chronic plaque PsO (e.g., pustular PsO, palmoplantar pustulosis, acrodermatitis of Hallopeau, erythrodermic, or guttate PsO) - Patients must not have had a history of current drug-induced PsO or a drug-induced exacerbation of pre-existing PsO - History of active ongoing inflammatory skin diseases other than PsO and PsA that could interfere with the assessment of PsO (eg, hyperkeratotic eczema) - History of severe renal insufficiency defined as creatinine clearance < 30 mL/min and/or requiring haemodialysis or peritoneal dialysis - History of clinically significant (per investigator's judgement) drug or alcohol abuse within the last 6 months - History of an allergic reaction or significant sensitivity to constituents of the study drugs (and its excipients) and/or other products in the same class - Patient must not have had major surgery performed within 12 weeks prior to randomisation or planned during the conduct of the study (e.g., hip replacement, aneurysm removal, stomach ligation) - No known active severe acute respiratory syndrome coronavirus 2 (SARS-CoV-2) infection. If a patient had signs/symptoms suggestive of SARS-CoV-2 infection, they would undergo molecular (e.g., polymerase chain reaction [PCR]) testing to rule out SARS-CoV-2 infection. In addition, if based on the answers to the SARS-CoV-2 Infection Risk Assessment Tool the site considered the patient currently at risk for developing SARS-CoV-2 infection, then the patient was either tested or advised to come back for study screening after 14 days. Patients who did not meet SARS-CoV-2 infection eligibility criteria were screen failed and only rescreened after they met the following SARS-CoV-2 infection viral clearance criteria: At least 14 days since first PCR test result had passed in asymptomatic patients or 14 days since recovery, defined as resolution of fever without use of antipyretics and improvement in symptoms - Patients without evidence of either Hepatitis B virus or hepatitis C virus infection - Patients must not have had any of the following medical diseases or disorders: - Recent (within past 6 months) cerebrovascular accident or myocardial infarction - History of an organ transplant which requires continued immunosuppression - Active or suspected malignancy or history of any malignancy within the last 5 years except for successfully treated non-melanoma skin cancer or localised carcinoma in situ of the cervix - Prior history of suicide attempt at any time in the patient's lifetime prior to signing the informed consent and randomisation, or major depression or suicidal ideation or attempt requiring hospitalisation within the last 3 years prior to signing the informed consent - Hereditary problems of galactose intolerance, total lactase deficiency, or glucose-galactose malabsorption - Patient must not have had concurrent clinically significant medical conditions other than the indication being studied or any other reason that the investigator determines would interfere with the patient's participation in this study, would make the patient an unsuitable candidate to receive study drug, or would put the patient at risk by participating in the study - Patients must not have had any prior exposure to risankizumab or apremilast - Patient must not have received any live viral or bacterial vaccine within 4 weeks prior to the first dose of study drug or expect the need for live vaccination during study participation including at least 140 days (20 weeks or as guided by the local risankizumab label [if approved], whichever is longer) after the last dose of risankizumab or at least 28 days after the last dose of apremilast - Patient did not receive any systemic biologics or photochemotherapy to treat PsO prior to the Baseline visit - Patient must not have been treated with any investigational drug within 30 days or 5 half-lives of the drug (whichever is longer) prior to the first dose of study drug or be currently enrolled in another interventional clinical study - Patient did not receive for at least 14 days prior to baseline any topical treatment for PsO or any other skin condition (including, but not limited to: e.g., corticosteroids, vitamin D analogues, vitamin A analogues, pimecrolimus, retinoids, salicyl vaseline, salicylic acid, lactic acid, tacrolimus, tar, urea, or anthralin) - Patient were not treated with any strong cytochrome P450 enzyme inducers (e.g., rifampin, phenobarbital, carbamazepine, phenytoin, St. John's Wort) within 30 days or 5 half-lives of start of treatment with apremilast |

Table S2 Description of adverse events in the IMMpulse study

| **Study Period** | **Description** |
| --- | --- |
| TEAEs in Period A | TEAEs in Period A were defined as any AE with an onset date on or after the first dose of the study drug in Period A and before the first dose of the study drug in Period B. If no study drug was administered in Period B, TEAEs in Period A included any AE with an onset date within 140 days of the last dose of risankizumab in Period A and within 28 days of the last dose of apremilast in Period A. |
| TEAEs in Period B | TEAEs in Period B were defined as any AE with an onset date on or after the first dose of study drug in Period B, and within 140 days of the last dose of risankizumab and 28 days of the last dose of apremilast in Period B. A TEAE during the administration of risankizumab (ie all risankizumab exposure) was defined as any AE with an onset date on or after the first dose of risankizumab and within 140 days after the last dose of risankizumab. |
| Serious AEs | Serious AEs were defined as any AE meeting one or more criteria: death, life-threatening, significant, or persistent disability/incapacity, congenital anomaly/birth defect (including fetal loss), important medical event, and initial inpatient hospitalisation or prolongation of hospitalisation. |

AE. Adverse events; TEAE, treatment-emergent adverse events

Table S3 Summary of week 52 efficacy among all apremilast-treated patients at baseline who achieved PASI 75 response at week 16 and switched to either risankizumab or continued apremilast (weeks 16 ─ 52)

|  | Apremilast switched to risankizumab  (N = 20)  n (%), (95% CI) | Continued apremilast  (N = 22)  n (%), (95% CI) |
| --- | --- | --- |
| **Clinical outcomes** |  |  |
| PASI 90 | 14 (70.0), (49.9, 90.1) | 3 (13.6), (0.0, 28.0) |
| sPGA 0/1 | 15 (75.0), (56.0, 94.0) | 10 (45.5), (24.6, 66.3) |
| PASI 75 | 18 (90.0), (76.9, 100.0) | 12 (54.5), (33.7, 75.4) |
| PASI 100 | 11 (55.0), (33.2, 76.8) | 2 (9.1), (0.0, 21.1) |
| sPGA 0 | 11 (55.0), (33.2, 76.8) | 2 (9.1), (0.0, 21.1) |
| **Patient-reported outcomes** |  |  |
| PSS 0/1 | 13 (65.0), (44.1, 85.9) | 2 (9.1), (0.0, 21.1) |
| DLQI 0/1 | 13 (65.0), (44.1, 85.9) | 3 (13.6), (0.0, 28.0) |

CI, confidence interval; DLQI, Dermatology Life Quality Index; PASI, Psoriasis Area and Severity Index; sPGA, static Physician's Global Assessment; PSS, Psoriasis Symptoms Scale

Period B ITT responder population included patients who were randomised to apremilast in Period A, achieved PASI 75 at week 16 and either switched to risankizumab or continued with apremilast in Period B.

Non-responder imputation incorporated multiple imputations to handle missing data only due to COVID-19.

Table S4 Treatment-emergent adverse events of special interest in the IMMpulse study

|  | **Period A**  **Baseline to week 16**  **All patients randomised at baseline** | | | | **Period B**  **Week 16 to week 52**  **All patients randomised to apremilast at baseline** | | | |
| --- | --- | --- | --- | --- | --- | --- | --- | --- |
|  | **Risankizumab**  (N = 118)  n (%) | **Risankizumab**  (N = 118)  (PYs = 35.8)  (E/100PYs) | **Apremilast**  (N = 234)  n (%) | **Apremilast**  (N = 234)  (PYs = 67.3)  (E/100PYs) | **Apremilast/**  **risankizumab**  (N = 102)  n (%) | **Apremilast switched to**  **risankizumab**  (N = 102)  (PYs = 86.9)  (E/100PYs) | **Continued**  **apremilast**  (N = 97)  n (%) | **Continued**  **apremilast**  (N = 97)  (PYs = 40.2)  (E/100PYs) |
| Adjudicated MACE | 0 | 0 | 1 (0.4) | 2 (3.0) | 0 | 0 | 0 | 0 |
| Extended MACE | 0 | 0 | 1 (0.4) | 2 (3.0) | 0 | 0 | 1 (1.0) | 1 (2.5) |
| Serious infections | 0 | 0 | 1 (0.4) | 1 (1.5) | 0 | 0 | 0 | 0 |
| Tuberculosis | 0 | 0 | 0 | 0 | 0 | 0 | 0 | 0 |
| Opportunistic infections excluding tuberculosis and herpes zoster | 0 | 0 | 0 | 0 | 0 | 0 | 0 | 0 |
| Injection site reactions | 2 (1.7) | 3 (8.4) | 0 | 0 | 2 (2.0) | 3 (3.5) | 0 | 0 |
| Malignancies | 0 | 0 | 0 | 0 | 0 | 0 | 1 (1.0) | 1 (2.5) |
| NMSC | 0 | 0 | 0 | 0 | 0 | 0 | 0 | 0 |
| Malignant tumour excluding NMSC | 0 | 0 | 0 | 0 | 0 | 0 | 1 (1.0) | 1 (2.5) |
| Hypersensitivity | 3 (2.5) | 3 (8.4) | 1 (0.4) | 2 (3.0) | 4 (3.9) | 11 (12.7) | 2 (2.1) | 2 (5.0) |
| Serious hypersensitivity | 0 | 0 | 0 | 0 | 1 (1.0) | 3 (3.5) | 0 | 0 |
| Adjudicated anaphylactic reaction | 0 | 0 | 0 | 0 | 0 | 0 | 0 | 0 |
| Hepatic events | 0 | 0 | 3 (1.3) | 4 (5.9) | 1 (1.0) | 1 (1.2) | 0 | 0 |

E, events; MACE, major adverse cardiovascular events; NMSC, non-melanoma skin cancer; PY, patient-years

Table S5 Summary of safety for all patients receiving risankizumab in IMMpulse

|  | **Risankizumab** N = 268 n (%) | **Risankizumab** N = 268 E (E/100 PY) PY = 241.0 |
| --- | --- | --- |
| **All AEs** | 167 (62.3) | 422 (175.1) |
| **AE with reasonable possibility of being related to study treatment** | 28 (10.4) | 40 (16.6) |
| **Severe AE** | 15 (5.6) | 19 (7.9) |
| **Serious AE** | 12 (4.5) | 17 (7.1) |
| **AE leading to discontinuation of study drug** | 1 (0.4) | 1 (0.4) |
| **AE leading to death** | 0 | 0 |

AE, adverse event; PY, patient-years
